# Development of an innovative clinical pharmacy service in a urology surgical unit: a new initiative from Qatar

**DOI:** 10.1080/20523211.2024.2401478

**Published:** 2024-09-23

**Authors:** Lina Naseralallah, Somaya Koraysh, Nour Isleem, Afif Ahmed, Moza Al Hail

**Affiliations:** Pharmacy Department, Hamad Medical Corporation, Doha, Qatar

**Keywords:** Clinical pharmacy, surgical pharmacy, pharmacist interventions, urology, surgery, medication safety

## Abstract

**Purpose:**

To provide an insight into the role of a clinical pharmacy initiative in a surgical urology unit through evaluating the nature, significance, associated medications, and acceptance rate of pharmacist interventions.

**Methods:**

A cross-sectional study was carried out at the Ambulatory Care Center (ACC), Doha, Qatar. Data related to clinical pharmacist interventions and associated rationale were classified according to the nature of the intervention using an adapted classification system. The assessment of the severity followed the National Patient Safety Agency (NPSA) Risk Matrix. Linear regression, Kruskal–Wallis, and post-hoc analyses were performed to determine the association between patient-related and medication-related characteristics on pharmacist interventions.

**Results:**

A total of 3284 interventions (on 1486 patients) were analysed. Most patients (*n* = 1105; 74.4%) had 1–2 interventions. Age and gender showed a positive linear correlation with the number of interventions per patient (*p* < 0.01). Majority of interventions were related to pharmacological strategy (*n* = 1858; 56.6%) and quantity of drug (*n* = 821; 25%). Additional drug therapy (*n* = 748; 22.78%) was the most common subcategory followed by optimum dose/frequency (*n* = 691; 21.04%) and discontinuation of medications (*n* = 352, 10.72%). Anti-infectives were the most identified drug category (*n* = 798, 55.1%). Most interventions (59.4%) were of moderate significance; patients with moderate interventions were found to be older compared to patients with minor interventions (*p* = 0.032). Prescribers’ acceptance rate was high (>90%), with a notable increase of 6.6% from 2021 to 2023.

**Conclusion:**

This study showed that the clinical pharmacy service in the urology surgical field was a fruitful initiative. The clinical pharmacist's role has expanded to include not only therapeutic optimisation while ensuring medication safety across the continuum of perioperative care but also the identification and management of untreated health problems. The dynamic and complexity of the urology patient population challenge clinical pharmacists; however, the practice concepts remain the same as in any other clinical setting.

## 1. Introduction

Pharmacists are placed in an ultimate position within the health system, allowing them to oversee almost all medication processes and promote rational medication usage (Alzahrani et al., 2021; Hooper et al., 2009; Naseralallah, Al-Badriyeh, et al., 2023). With their constantly expanding role owing to their specialised knowledge and expertise, pharmacists have been recognised as key players in the pharmaceutical care service plans. Multiple studies have elucidated the positive impact of pharmacist’s involvement and interventions on multiple important outcomes; namely, reduction in preventable adverse drug events, mortality rates, length of hospital stay, drug-related re-admissions, and treatment costs (Abushanab et al., 2023; Butt et al., 2019; Elnour et al., 2022; Graabaek & Kjeldsen, 2013; Ravn-Nielsen et al., 2018; Saha et al., 2019; Skjot-Arkil et al., 2018).

Within hospital settings, surgical departments are one of the main areas where medication therapy management is essential, owing to the need for medical perioperative care for all admitted patients. Multiple drug therapy aspects are involved in the perioperative medical care, including perioperative chronic medications management, pain management, anti-infectives, anticoagulation, fluid management, vitals management, as well as other aspects pertaining to patient’s needs perioperatively (Lovely et al., 2019; Mohammed et al., 2022). It has been reported that up to 69.5% of elective surgery patients are at risk of at least one drug-related problems (DRP) during their hospital stay (Mohammed et al., 2022). Moreover, it has been reported that medication discrepancies are more frequent in surgical patients compared to medical patients, owing to the inappropriate management of home medications perioperatively (Almeida et al., 2017; Mohammed et al., 2022). The incorporation of multidisciplinary surgical teams where pharmacists are actively involved in medication therapy management has shown its merits in reducing errors, medications discrepancies, inappropriate prescribing behaviour, as well as optimising patient-centred care including improving therapeutic and clinically important outcome (Naseralallah et al., 2024a, 2024b; Naseralallah et al., 2024).

In line with the global evolving nature of the pharmacy profession, the Pharmacy Department at Hamad Medical Corporation (HMC) has always strived to upgrade its services to meet the institution and national vision (Hamad Medical Corporation, 2024). Clinical pharmacy is one of the cardinal services provided by HMC’s pharmacy department. As part of their continuous efforts for improvement, HMC pharmacy department has taken the initiative to start a clinical pharmacy service in a Urology Surgery Department in the Ambulatory Care Center (ACC) in 2020, whereby a dedicated clinical pharmacist is integrated into the surgical urology team to oversee all medication-related processes to ensure optimum pharmaceutical care that delivers the desired health outcomes. All interventions done by the clinical pharmacist are documented electronically through the patient record system (Cerner, 2022).

The incorporation of a clinical pharmacist in surgical urological departments was viewed as necessary due to the increasing complexity and seniority of admitted patients. The surgical urology unit encompasses a wide range of procedures with variable complexities (e.g. invasive or minimally invasive) which require different perioperative care. Additionally, surgical urology departments are becoming increasingly affected by the growing demographic of older population worldwide (Drach & Griebling, 2003; Kabarriti et al., 2016). Geriatric patients often present with a multitude of chronic diseases and polypharmacy (Mohammed et al., 2022; Umemura et al., 2019), which demands a special consideration in medication management perioperatively. Only one study has been identified to focus on the impact of integrating pharmacists in urology teams. Findings from this study showed that pharmacist interventions were associated with an improved urinary function status (*p* = 0.022) of the included patients (Umemura et al., 2019). Nevertheless, to date there are no published studies that examined and analysed pharmacist interventions in urology settings. Given the novelty of this service and the lack of informing literature on the role of pharmacists in this department, it is imperative to evaluate and analyse clinical pharmacists interventions to obtain an insight on the current status of clinical pharmacists involvement and to explore possible areas of improvement (Albayrak et al., 2022; Deawjaroen et al., 2022; Lekpittaya et al., 2024). The aim of this study is to quantify, classify, and analyse the interventions provided by the surgical urology pharmacist and provide an insight on the evolvement of practice since the initiation of the service.

## 2. Materials and methods

### 2.1. Ethics approval

The study was granted ethics approval by the Medical Research Center (MRC), HMC in 2024 (MRC-01-24-111). As the study was a retrospective chart review, informed consent requirement was waved, and no further ethical approval was required. Anonymity of data was maintained through the study as data were collected and maintained confidentially, with usage of codes to mask possible identifiers.

### 2.2. Study design and setting

This cross-sectional study was carried out at the ACC, Doha, Qatar. ACC is an innovative facility under HMC that opened in 2017 and it offers a new approach to surgical care in Qatar using advanced clinical and surgical practices.

In October 2020, the Pharmacy Department in collaboration with the Urology Department launched a clinical pharmacy service in the inpatient urology unit in ACC. The clinical pharmacy service consists of one clinical pharmacist covering the 24-bed unit and provides ward coverage 5 days a week (Sunday to Thursday). The clinical pharmacist follows up with patients from admission until the point of discharge and they perform multiple activities in collaboration with the healthcare team including attending rounds, medicines optimisation, medicines reviews, reconciliation, and responding to drug information inquiries. On weekends, medication verification was provided by the unit dose pharmacy, however, no clinical pharmacy services were available.

### 2.3. Data processing

For the purpose of this study, ‘any professional activity by the pharmacists directed towards improving the quality use of medicines and resulting in a recommendation for a change in the patient's medication therapy, means of administration or medication-taking behavior' are regarded as clinical interventions (Pharmacy, 2005). Clinical pharmacists submit their interventions electronically through an ad-hoc note embedded on the electronic medical records (EMR). All interventions that took place in the urology ward during a 3-year period (January 2021 to January 2024) were retrieved by an information technology pharmacist. The first 3 months after implementing the service were excluded as they were orientation and introduction of the service. No restriction on age, gender, or any patient characteristics was imposed. The acquired data were processed on Microsoft Excel in an anonymous form. Data cleaning was used to eliminate duplicated records, incomplete or unclear cases. Categorisation was conducted primarily by one author (LN) followed by independent checks by a second author (SK).

Data related to pharmacist interventions and associated rationale were quantified and classified according to the nature of the intervention. The classification system used was adapted from previous studies with modifications to capture the common reoccurring interventions (Faus et al., 2007; Haque et al., 2021). Interventions were categorised into five groups: (1) pharmacological strategy; (2) quantity of drug; (3) monitor; (4) documentation; and (5) drug information. Each group was further classified into sub-categories (e.g. optimum dose or frequency under quantity of drug) (Faus et al., 2007; Haque et al., 2021).

The medications associated with interventions were grouped into 16 classes: anti-infective agents, cardiovascular drugs, endocrine system and hormonal agents, gastrointestinal drugs, analgesics/anti-inflammatory drugs, anti-neoplastic and immunosuppression, blood derivatives and immunoglobulins, central nervous system agents, fluids and electrolytes, vitamins and nutritional agents, eye, ear, nose and throat (EENT) drugs, musculoskeletal and joint disease drugs, respiratory tract agents, urinary-tract disorders agents, total parenteral nutrition (TPN), and vaccines.

The significance of the interventions was graded on a scale of 1–5 based on the consequences that might arise from the risk. This scoring system was adapted from the National Patient Safety Agency (NPSA) Risk Matrix (A risk matrix for risk managers, 2008). A score of 1 refers to intervention with no or minimal clinical impact and a score of 5 indicates an intervention that could potentially prevent an organ- or life-threatening event (Table 1) (MacTavish et al., 2019).

**Table 1.** Intervention severity scale.

Severity level	1	2	3	4	5
Domain	Negligible	Minor	Moderate	Major	Catastrophic
Clinical impact	No clinical impact	Minimal clinical impact	Increase therapeutic benefit/avoidance of significant adverse event	Prevent serious therapeutic failure/ avoidance of serious adverse event	Prevent organ- or life-threatening event

Clinical pharmacists also record the outcome of the interventions (acceptance or rejection by the surgeon).

### 2.3. Data analysis

The data collected were analysed using SPSS software (version 22.0) and presented as frequencies, means and standard deviation (SD) as appropriate.

Linear regression was performed to determine the association between patient-related and medication-related characteristics on the number of pharmacist interventions; 2-sided *P*-values <0.05 were considered statistically significant. The Kruskal–Wallis test was applied to determine the influence of patient-related and medication-related characteristics on the significance of interventions. A post-hoc analysis utilising Bonferroni test for multiple analyses was used for any significant finding from the Kruskal–Wallis test.

## 3. Results

### 3.1. Characteristics of clinical pharmacists’ interventions

Characteristics of included patients are illustrated in Table 2. In this study, 1486 patients were included, 73% of which were males. Median age of included patients was 57 [interquartile range (IQR) 24] years. Following retrieval of data, a total of 3506 pharmacist interventions were identified over a 3-year period. A total of 222 intervention cases were excluded from the study as information was unclear or incomplete, or they were not for urology patients. The remaining 3284 submitted interventions formed the data sample included in this study.

**Table 2.** Characteristics of included patients.

Age	*Median (IQR)*^a^	57 (24)	
Gender	*Male*	1098	73.9%
	*Female*	388	26.1%
Number of interventions per patient	*Median (IQR)*^a^	1 (2)	
	*1–2 per patient*	1105	74.4%
	*3 or more per patient*	381	25.6%
Number of patients (*n* = 1486)	*2021*	477	30.8%
	*2022*	466	30.1%
	*2023*	543	35.1%
Number of interventions (*n* = 3284)	*2021*	997	30.4%
	*2022*	1020	31.1%
	*2023*	1267	38.6%

*^a^*Not normally distributed.

The number of reported interventions increased gradually over the study duration from 997 in 2021 to 1267 in 2023. A median of 1(2) interventions were recorded per each patient. In particular, 1105 (74.4%) of patients had 1 or 2 pharmacy interventions directed at their treatment plans, while 381 (25.6%) had 3 interventions or more. Both age (*R*^2^ = 0.023) and gender (*R*^2^ = 0.023) had a direct positive linear significant correlation with the number of reported interventions per patient; specifically, for every year increase in age, interventions per patient increased by 0.02, while being a female increased the number of interventions per patient by 0.679 compared to males.

### 3.2. Classification of interventions

Types and subtypes of interventions alongside reasons behind the interventions and clinical examples are described in Table 3. More than half of the interventions (56.6%) were addressing pharmacological strategies (*n* = 1858), followed by drug quantity (*n* = 821, 25%), monitoring of labs and diagnostics (*n* = 314, 9.6%), drug information inquiries (278, 8.5%), and incomplete documentation (*n* = 13, 0.4%).

**Table 3.** Overview of pharmacists’ interventions.

Intervention category	Subcategory	*n*	%	Reason for intervention	*n*	%	Examples
Pharmacological strategy (*n* = 1858, 56.6%)	Additional Therapy Required	748	22.78%	Untreated condition	323	43.18%	Anaemia (*n* = 129), dyslipidaemia (*n* = 63), diabetes (*n* = 52), hypertension (*n* = 37), bone and mineral disease (*n* = 18)
				Inpatient complications	231	30.88%	Electrolyte imbalance (*n* = 127), hyperglycaemia (*n* = 68), infection (*n* = 11), cardiac (*n* = 4)
				Inpatient management	85	11.36%	Diabetes (*n* = 75), Anticoagulation (*n* = 10)
				Prevention/prophylaxis	69	9.22%	VTE prophylaxis (*n* = 44), stress ulcer prophylaxis (*n* = 24), antibiotics (*n* = 1)
				Uncontrolled condition	40	5.35%	Hypertension (*n* = 23), diabetes (*n* = 17)
	Discontinue medication	352	10.72%	Duplicate therapy	135	38.35%	
				Not indicated	68	19.32%	Discontinue nifedipine as patient is not hypertensive
				No longer needed	41	11.65%	Completed treatment duration for fluconazole
				Kidney dysfunction	39	11.08%	Discontinue nephrotoxic medications (ibuprofen)
				Drug–Drug Interaction	30	8.52%	Discontinue tamsulosin as patient is on alfuzosin
				Adverse Drug Event	18	5.11%	Discontinue dapagliflozin due to recurrent UTIs
				Liver	12	3.41%	Discontinue simvastatin due to elevated LFTs
				Drug–Disease Interaction	4	1.14%	Delay due to active infection
				Optimise management	2	0.57%	
				Pregnancy	2	0.57%	
				According to indication	1	0.28%	
	Alternative Therapy	300	9.14%	Optimise management	91	30.33%	Switch cough suppressant to mucolytic for productive cough
				Culture	89	29.67%	Ceftriaxone resistant *E. coli*, switched to ertapenem
				Availability of product	25	8.33%	Bromazepam (non-formulary) to lorazepam
				Kidney dysfunction	20	6.67%	Shift dalteparin to heparin due to CrCl <10 ml/min
				Perioperative care	16	5.33%	Shift oral antidiabetics to insulin preoperatively
				Adverse Drug Event	13	4.33%	lisinopril induced cough, switched to amlodipine
				Drug–Drug Interaction	13	4.33%	Voriconazole and atorvastatin, shifted to rosuvastatin
				Inpatient complication	13	4.33%	Hyperglycaemia (*n* = 10), hypoglycaemia (*n* = 3)
				Infection signs	10	3.33%	
				Others	10	3.33%	
	Hold/Resume	265	8.07%	Omitted home medication	83	31.32%	Resume perindopril due to elevated BP
				Home medications, after surgery	76	28.68%	
				Inpatient complication	36	13.58%	Hypotension (*n* = 21), hypoglycaemia (*n* = 13), bradycardia (*n* = 1)
				Perioperative care	35	13.21%	
				Kidney dysfunction	17	6.42%	Hold valsartan
				Adverse drug event	9	3.40%	
				Liver	3	1.13%	
				Not indicated	2	0.75%	
				Drug interactions	3	1.13%	
				Inpatient management	1	0.38%	Anticoagulation
	Formulation Selection	102	3.11%	IV to oral	76	74.51%	Ceftriaxone to cefixime
				Oral to IV	8	7.84%	Iron supplementation PO to IV
				interchanging oral dosage forms (Tab, susp, NGT)	8	7.84%	Amlodipine tablet to suspension as patient has dysphagia
				IV to other (non-oral)	3	2.94%	
				Enteral feeding	3	2.94%	
				Availability of product	2	1.96%	
				Per guidelines/hospital protocol	2	1.96%	
	Lifestyle modifications	91	2.77%	Untreated condition	91		Prediabetes (*n* = 82), dyslipidaemia (*n* = 4), diabetes (*n* = 3), hypertension (*n* = 2)
Quantity of drug (*n* = 821, 25%)	Optimum Dose	515	15.68%	Optimise management	235	45.63%	Vancomycin dose for severe *C. difficile* is 125 mg PO q6h
				Kidney dysfunction	149	28.93%	Reduce levofloxacin dose to 250 mg q24 h (CrCl 45 ml/min)
				Weight	87	16.89%	Enoxaparin dose increased to 60 mg due to BMI >40
				Kidney (unnecessary dose adjustment)	18	3.50%	Increase ertapenem dose to 1 g daily as CrCl > 30 ml/min
				Age	13	2.52%	Reduce ceftriaxone to 1 g/dose for a 14-year-old patient
				Liver	9	1.75%	
				Platelet	4	0.78%	
	Optimum Frequency	176	5.36%	Optimise management	97	55.11%	Reduce esomeprazole to once daily
				Liver	34	19.32%	Reduce paracetamol from q6 h to q8h
				Kidney dysfunction	24	13.64%	Reduce daptomycin frequency to q48 h (CrCl 22 ml/min)
				Per guidelines/hospital protocol	18	10.23%	Amlodipine to once daily
				Kidney (unnecessary dose adjustment)	3	1.70%	
	Inappropriate Duration	76	2.31%	According to indication	62	81.58%	Renew piperacillin-tazobactam order to complete a 14-day treatment course
				Per guidelines/hospital protocol	9	11.84%	Discontinue ketorolac after 5 days of treatment
				Drug–Drug Interaction	2	2.63%	
				Others	3	3.95%	
	Optimum Administration	54	1.64%	Optimise management	26	48.15%	Shift paracetamol from PRN to routine due to increased pain post-operatively
				Per guidelines/hospital protocol	25	46.30%	Add numerical pain score to analgesics orders
				Drug–Drug Interaction	2	3.70%	
				Inpatient complication	1	1.85%	Hypotension
Monitor (*n* = 314, 9.6%)	Appropriate Laboratory Recommended	298	9.07%	Labs for diagnosis	217	73.31%	HbA1c (*n* = 70), lipid panel (*n* = 62), electrolytes (*n* = 34), iron panel (*n* = 12), glucose (*n* = 12), Anaemia workup (*n* = 9), thyroid function (*n* = 16)
				Labs for kidney/liver function	57	19.26%	Renal function tests (*n* = 22), liver function tests (*n* = 32)
				Labs for drug levels	15	5.07%	Trough levels (vancomycin, gentamicin, cyclosporine) (*n* = 9), INR (*n* = 6)
				Labs for drug–drug interactions	9	3.04%	Trimethoprim-sulfamethoxazole and phenytoin, to follow phenytoin levels
	Appropriate Procedure Recommended	16	0.49%	Consultation request	8	50.00%	Endocrine consult for persistent hypoglycaemia
				ECG	7	43.75%	Bed aquiline and levofloxacin
				Culture	1	6.25%	
Drug Information (*n* = 278, 8.47%)	Drug Information Inquiry	278	8.47%	Optimum dose (kidney)	82	29.50%	Optimising dose of ampicillin-sulbactam for a patient on peritoneal dialysis
				Optimum Administration	70	25.18%	To administer amlodipine only if SBP > 145
				Allergy	29	10.43%	Inquiring about cross-allergy between cephalosporin and clindamycin
				Medication selection	29	10.43%	
				Anticoagulation	17	6.12%	Perioperative heparin bridging management
				Medication selection and dosing	11	3.96%	Antibiotic choice and dosing at discharge
				Pregnancy	10	3.60%	Use of NSAIDs in third trimester
				Optimise management	8	2.88%	
				Adverse Drug Event	6	2.16%	Pedal oedema due to amlodipine
				Others	10	3.60%	
Incomplete prescriptions (*n* = 13, 0.4%)	Missing Weight	13	0.40%				

#### 3.2.1. Pharmacological strategy

Pharmacists had an active role in adjusting and contributing to pharmacological strategies. The most common form of contribution was through recommending an additional therapy to the treatment plan (*n* = 748, 40.3%), mainly to target untreated (*n* = 323, 43.18%) or uncontrolled conditions (*n* = 40, 5.35%), inpatient complications (*n* = 231, 30.88%), inpatient management of chronic diseases (*n* = 85, 11.36%), or adding a prophylactic therapy such venous thromboembolism (VTE) prophylaxis or stress ulcer prophylaxis (*n* = 69, 9.22%).

Additionally, pharmacists had a role in discontinuing medications (*n* = 352, 18.9%) mainly due to the following reasons: therapy duplication (*n* = 135, 38.35%), medication without indication (*n* = 68, 19.32%), finished treatment duration (*n* = 41, 11.65%), kidney dysfunction (*n* = 39, 11.08%), or drug–drug interactions (*n* = 30, 8.52%).

Recommending an alternative therapy was another subtype of the clinical pharmacist interventions (*n* = 300, 16.2%). The rationale behind the change was mainly to optimise the treatment plan (*n* = 91, 30.33%) or adjusting treatment based on culture results (*n* = 89, 29.67%). To elaborate, pharmacists recommended de-escalating and escalating antimicrobial therapy in 40 and 49 cases based on culture results, respectively.

Another subtype of the pharmacist intervention was through holding (*n* = 103, 5.5%) or resuming (*n* = 162, 8.7%) medications. The main reason for resuming medications was omitted home medications on admission (*n* = 83, 31.32%), while inpatient complications (*n* = 36, 13.58%) and perioperative management (*n* = 35, 13.21%) were the main reasons for holding medications.

Other interventions included adjustment of formulation selection (*n* = 102, 5.4%), mostly shifting from intravenous (IV) to oral route (*n* = 76, 74.51%), and lifestyle modifications (*n* = 91, 4.9%), primarily due to the identification of prediabetes patients (*n* = 82, 90%).

#### 3.2.2. Quantity of drug

This type of intervention was the second most reported by pharmacists. It comprised intervening to optimise drug dose (*n* = 515, 62.7%), frequency (*n* = 176, 21.4%), duration (*n* = 76, 9.3%), and administration schedule (*n* = 54, 6.6%) (Table 3). Optimising inpatient management was the main reason for intervening on medication dosing (*n* = 235, 45.63%), frequency (*n* = 97, 55.11%), and administration (*n* = 26, 48.15%). Renal and hepatic impairment were also important causes for adjusting the dose (*n* = 149, 28.93% and *n* = 9, 1.75%) and frequency (*n* = 24, 13.64% and *n* = 34, 19.32%). It was noted that pharmacists intervened to correct an unnecessary dose or frequency adjustment due to kidney function in 18 (3.5%) and 3 (1.7%) of the cases, respectively.

Lastly, pharmacists intervened to adjust an inappropriate treatment duration (*n* = 76, 2.31%) mainly to comply with durations needed for each medical indication (*n* = 62, 81.58%), as in prolongation or cessation of antibiotic prescriptions for complete cure of infections (Table 3). To elaborate, pharmacists decreased the duration of regimens in 55 cases and increased the duration in 21 cases.

#### 3.2.3. Monitor of labs and diagnostics

Pharmacists requested labs and diagnostics for 9.6% (*n* = 314) of admitted patients. Most interventions pertaining to monitoring revolved around laboratory follow up, especially for diagnosing or following up on certain disease states (*n* = 217, 73.3%), liver or kidney function tests (*n* = 57, 19.26%), drug interactions (*n* = 9, 3.04%), or trough levels (*n* = 15, 5.07%).

Other recommended procedures included electrocardiogram (ECG) (*n* = 7, 43.75%) or culture (*n* = 1, 6.25%), or requesting specialty team consultation such as endocrine (*n* = 8, 50%).

#### 3.2.4. Drug information inquiries

During the study period, the clinical pharmacist responded to 278 (8.47%) drug information questions. The most common causes for inquiries were related to the optimum dose according to creatinine clearance (*n* = 82, 29.5%) by the physicians and optimum administration of medications (*n* = 70, 25.1%) by the nurses.

Inquiring about allergies, especially cross-allergy between antibiotics, was sought in 10.43% of interventions (*n* = 29). Medication selection encompassed 39 interventions (14.39%). Other reasons for drug information inquiries included anticoagulation scheduling perioperatively (*n* = 17, 6.12%), safety of medications in pregnancy (*n* = 10, 3.6%), and adverse drug events (*n* = 6, 2.16%).

### 3.3. Drug categories associated with intervention

**Figure 1.**
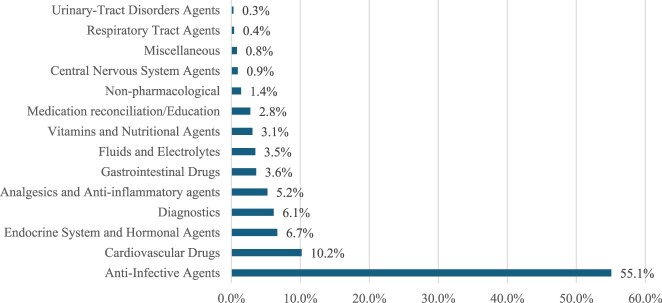
Drug categories targeted in clinical pharmacists’ interventions.

Drug categories targeted in the interventions are depicted in Figure 1 (for detailed description, see online Supplemental Material Table S1). As multiple interventions targeted multiple agents (i.e. multiple drugs per a single reported intervention), it was expected that the total number of implicated drugs would not match the total number of interventions. Most interventions were on anti-infective agents (*n* = 798, 55.1%), namely antibiotics (*n* = 739, 93%). Cardiovascular drugs were targeted in 10.2% (*n* = 743) of documented interventions, namely anticoagulants (*n* = 333, 45%) and antihypertensives (*n* = 304, 41%). Endocrine and hormonal agents, namely antidiabetics (*n* = 452, 93%), were targeted in 486 (6.7%) of interventions. Pharmacists’ interventions also involved other aspects, including diagnostics (*n* = 448, 6.1%) and non-pharmacological interventions (*n* = 103, 1.4%). Lab tests (*n* = 391, 87%) were the most frequently requested interventions under diagnostics, while non-pharmacological recommendations were mainly lifestyle modifications (*n* = 98, 95%).

### 3.4. Significance of interventions

**Figure 2.**
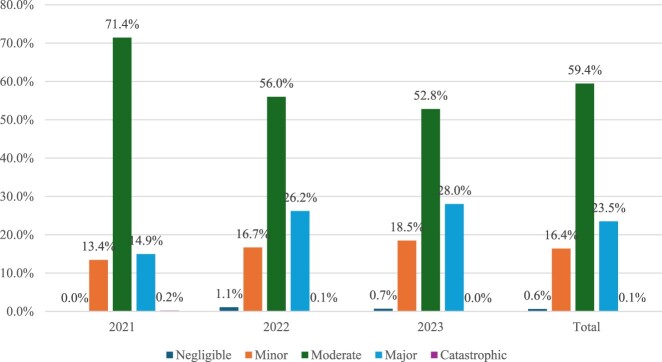
Severity of clinical pharmacists’ interventions.

The significance level of interventions is presented in Figure 2. Clinical examples of different levels of significance are reported in Supplemental Material Table S2. A pattern of increased focus on reporting major interventions has been noted, from comprising 14.9% of interventions in 2021 to 28% in 2023. Simultaneously, a slight decline in the number of moderate interventions has been recorded (from 71.4% in 2021 to 52.8% in 2023).

A total of 1952 of the interventions (59.4%) were classified as moderate in nature (related to endocrine agents (*n* = 406, 21%), cardiovascular drugs (*n* = 349, 18%), and lab tests (*n* = 337, 17%)). Second most reported significance level was major interventions (*n* = 771, 23%), mainly targeting anti-infective agents (*n* = 411, 53%) and cardiovascular agents (*n* = 201, 26%). A quarter of the interventions were described as minor (*n* = 538, 16%); mostly on vitamins and nutritional agents (*n* = 172, 32%), followed by analgesics (*n* = 108, 20%), and non-pharmacological recommendations (*n* = 16%). All negligible interventions were related to analgesics (*n* = 20, 1%), mainly due to missing numerical pain score. Only three interventions were described as catastrophic, two out of three were related to anti-infective medications. The first one recommended switching to vancomycin prior to surgery due to identifying a Methicillin-resistant *Staphylococcus aureus* (MRSA) infection which led to delaying the surgery to avoid urinary sepsis. The second one recommended termination of teratogenic medication in a pregnant patient. The third life-threatening intervention was related to anticoagulants use (enoxaparin, rivaroxaban) and duration of therapy in a high-risk patient which could have led to VTE event.

Analysis of the distribution of age across the different categories of interventions significance indicated the presence of significant difference between the different categories (Kruskal–Wallis; *p* = 0.017). A post-hoc analysis identified the significant difference to be between minor and moderate interventions, with moderate interventions group having older patients compared to minor interventions group (*p* = 0.032).

### 3.5. Prescribers’ response

**Figure 3.**
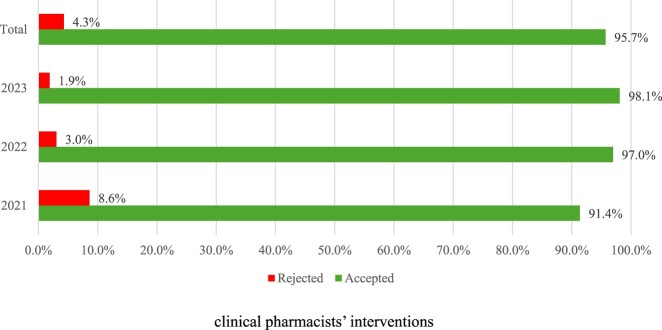
Prescribers’ responses to clinical pharmacists’ interventions.

Pharmacists’ interventions appear to be well received by prescribers as the overall acceptance rate was 95.7% during the study period (Figure 3). An increase in acceptance rate was noted as the years progressed (from 91.4% to 98%).

## 4. Discussion

### 4.1. Key findings

A total of 3284 clinical pharmacists’ interventions were included and analysed in this study, with a steady increase over the study period. Our findings show that the majority of pharmacist interventions were related to changes in pharmacological strategy (*n* = 1858; 56.6%), followed by changes in quantity of drug (*n* = 821; 25%). Subtypes of interventions most frequently related to additional drug therapy (*n* = 748; 22.78%), optimum dose/frequency (*n* = 691; 21.04%), discontinuation of medication (*n* = 352; 10.72%), and alternative therapy (*n* = 300; 9.14%). Reasons behind the interventions were mainly untreated condition (e.g. newly diagnosed diabetes), altered kidney function, perioperative management of chronic conditions/medications (e.g. anticoagulants or antidiabetic drugs), and inpatient complications (e.g. electrolyte imbalance).

Anti-infective agents were involved in more than half of the interventions, followed by cardiovascular (10.2%) and endocrine medications (6.7%). Most interventions were of moderate severity (59.4%) with a considerable increase in the proportion of moderate interventions as compared to minor ones over the years. Similarly, a significant increase of 6.6% was observed in the acceptance rate of clinical pharmacists’ interventions between 2021 and 2023. Our statistical analyses showed that age and gender had a positive linear correlation with the quantity of interventions per patient. Likewise, patients with moderate interventions were older as compared to patients with minor interventions (*p* = 0.032).

The predominant clinical pharmacist intervention in our study was changes to the pharmacological strategy followed by quantity of the drug. The literature has inconsistent findings in relation to the most common type of clinical pharmacist intervention; however, this is understandable as the area of practice and patient populations mandate adaption by pharmacists and targeting of the most common DRPs that may occur in this area. For instance, in line with our findings, the most common pharmacist intervention in medical and general surgical units and in patients taking direct oral anticoagulant (DOAC) was changes to the pharmacological strategy (Haque et al., 2021; Naseralallah, Al-Badriyeh, et al., 2023; Ngige et al., 2018). Nonetheless, in neonatal intensive care unit (NICU), changes to the quantity of drug were more common (Naseralallah et al., 2020).

Our finding showed that implementing a clinical pharmacist in a urology surgical unit presents a unique opportunity to identify patients with untreated medical conditions (particularly anaemia, dyslipidaemia, diabetes, and hypertension) and initiate appropriate therapy. It also enabled the optimisation of known uncontrolled medical conditions. The American Society of Health System Pharmacists (ASHP) 2015 Initiative elucidates in the tenets for medication therapy management ‘performing or obtaining necessary assessments of the patient’s health status' and ‘monitoring and evaluating the patient’s response to therapy, including safety and effectiveness' (Myers, 2004). The Canadian Society of Hospital Pharmacists (CSHP) 2015 Initiative also contains nearly identical language in relation to pharmacotherapy management (Musing, 2008). Whilst clinical pharmacists in HMC do not have the authority to order laboratory tests, they can advise the physician to request tests where appropriate to facilitate drug therapy decision-making. Although there is a debate regarding pharmacists’ diagnostic role both within the pharmacy profession and between professions (mainly medicine and pharmacy), there is some evidence that pharmacists commonly engage in diagnosis (Chernushkin et al., 2012; Rutter, 2020). It is noteworthy that this role may not be maintained in other surgical specialties as patients admitted to urology are usually older adults which means that they are more prone to chronic conditions.

Adjusting the dose or frequency was found to be the second most common subtype of interventions. Numerous studies have identified dosing errors (primarily due to kidney dysfunction) as a leading contributory factor to mistakes across various practice areas (Al Rowily et al., 2022; Gates et al., 2019; Lewis et al., 2009; Naseralallah et al., 2022; Naseralallah, Stewart, et al., 2023; Thomas et al., 2019). This is especially dire in patients with urological disorders due to the interplay between chronic kidney disease (CKD) and urological conditions (e.g. urothelial carcinoma) (Chinnadurai et al., 2020; Han et al., 2016). At the same time, urologic diseases might worsen renal function by contributing to the development and progression of CKD or increasing the incidence of acute kidney injury (AKI) due to the common occurrences of obstructive uropathy and urosepsis, as well as the deterioration in renal function that sometimes follows renal surgeries (Caddeo et al., 2013; Lai et al., 2019). Altered kidney function was also a prevalent reason for multiple clinical pharmacists’ interventions in the current study, including alternative therapy or holding medications. This further underscores the potential impact of clinical pharmacists in urology department as a considerable number of patients might have fluctuating creatinine clearance which requires close monitoring by the clinical pharmacist and subsequent clinical judgment and decision-making.

Findings from our study showed that the clinical pharmacist played an integral role in optimising perioperative pharmacotherapy across the care continuum. This included a wide range of activities targeting management of chronic conditions (e.g. anticoagulation, diabetes, or hypertension,), inpatient complications (e.g. infections, electrolyte imbalance, pain, nausea and vomiting, nutrition management, or fluid management), as well as other aspects of medication therapy management. The integration of clinical pharmacist in enhanced recovery after surgery (ERAS) programs has been increasingly emphasised in recent years owing to the clinical pharmacists dedicated training and expertise which provides a unique perspective on the multidisciplinary perioperative teams which enhances the outcomes of ERAS pathways and subsequently overall patient surgical outcomes (AbuRuz et al., 2021; Lovely et al., 2019; Wireko et al., 2023; Xie et al., 2023).

In this evaluation, more than half of the interventions were linked to anti-infectives agents, this includes optimisation of both perioperative antibiotic prophylaxis and management of surgical site infections (SSI). This shed light on the potential roles clinical pharmacists could uptake in antimicrobial stewardship (AMS) programs in surgical settings. A plethora of studies have yielded encouraging outcomes in relation to the positive effect of pharmacist-led AMS programs in surgical settings (Abubakar et al., 2019; Elnour et al., 2022; Fésüs et al., 2021; Naseralallah et al., 2024a; Schroeder et al., 2022; Zhou et al., 2021). For instance, a study conducted in orthopaedic surgical unit showed that the involvement of clinical pharmacist in AMS programs led to a substantial improvement in adherence to surgical antibiotic prophylaxis guidelines, which was accompanied by decreased antibiotic exposure and cost (Fésüs et al., 2021).

### 4.2. Strengths and limitations

This research was performed in a teaching hospital with a high patient turnover rate. A large, comprehensive dataset was extracted over a substantial timeframe using sophisticated intervention reporting database. Additionally, we utilised a systematic and rigour approach to understanding and categorising clinical pharmacist interventions (Faus et al., 2007). However, classification using this taxonomy can be subjective particularly when there is a lack of adequate free text information available. Also, the documentation of interventions is mandatory according to HMC policy and is one of the objectives for the annual evaluation of clinical pharmacists; hence this might have led to overreporting of clinically irrelevant situations. Moreover, data was obtained from a single site in Qatar which might limit the generalisability. Lastly, the study did not document the consequences of clinical pharmacists’ interventions (i.e. the overall clinical outcomes).

### 4.3. Future directions

It is evident that surgical clinical pharmacists have a tremendous opportunity to improve clinical outcomes, particularly perioperative care. We therefore encourage pharmacists to pursue advanced clinical training and gather credentials that will give them added credibility to work in surgical settings, particularly urology. Additionally, nations should prioritise developing a career structure that supports specialisation and shaping of the surgical clinical pharmacist career. Health and academic institutions should allocate more funding to both undergraduate and postgraduate pharmacy programs to enable the incorporation of more surgical experiential learning opportunities in the pharmacy training curriculum. This will subsequently assist pharmacists in improving their clinical skills and confidence in clinical settings.

Future observational studies are needed to overcome the potential bias in relation to the method of reporting of pharmacist interventions. Qualitative studies (such as semi-structured interviews) of patients, nurses, surgeons, and pharmacists to further explore roles and impact of clinical pharmacist implementation in urology setting (and surgical settings in general) are required. Additional research should aim to extend the scope of this study to other surgical subspecialties to enable policymakers to judge the importance of initiating a clinical pharmacy service in each surgical subspecialty and triage its priority. This should also include the impact of these services on patient health outcomes and overall healthcare costs.

## 5. Conclusion

This study shows that providing clinical pharmacy services in the urology surgical field was a fruitful initiative at ACC. The clinical pharmacist's role has expanded to include not only therapeutic optimisation while ensuring medication safety across the continuum of perioperative care, but also the identification and management of untreated health problems. The dynamic nature and complexity level of the urology patient population challenge the clinical pharmacists; however, the practice concepts remain the same as in any other clinical setting. More research is needed to assess the long-term impact of this service as well as a thorough cost–benefit analysis as it is equally necessary for policymakers to consider to attain service excellence. Additional strategies such as strengthening clinical governance and pharmacists’ involvement in other surgical subspecialties are required to improve patient safety in surgical settings.

## Supplementary Material

Supplementary_material.docx
