# Supplementary material for: Development of an innovative clinical pharmacy service in a urology surgical unit: a new initiative from Qatar
Source: J Pharm Policy Pract. 2024 Sep 23;17(1):2401478. doi: 10.1080/20523211.2024.2401478 (PMC11421156; doi:10.1080/20523211.2024.2401478)
Supplement: Supplementary_material.docx [file JPPP_A_2401478_SM5495.docx]

| **Table S1. Drug categories (n=7295)** | |  |  |  |  |
| --- | --- | --- | --- | --- | --- |
| **Anti-Infective Agents** | **798** |  | **Cardiovascular Drugs** | **743** |  |
| *Antibiotics* | *739* | 93% | *HTN* | *304* | 41% |
| Penicillin | 108 | 15% | CCB | 149 | 49% |
| Cephalosporin | 256 | 35% | BB | 38 | 13% |
| Fluoroquinolones | 38 | 5% | ACE-i/ARB | 63 | 21% |
| Carbapenems | 161 | 22% | Others | 54 | 18% |
| Aminoglycosides | 21 | 3% | *Antiplatelet* | *33* | 4% |
| Glycopeptides | 32 | 4% | *Anticoagulant* | *333* | 45% |
| Others | 123 | 17% | *Cholesterol* | *73* | 10% |
| *Anti-fungal* | *48* | 6% | **Fluids and Electrolytes** | **253** |  |
| *Others* | *11* | 1% | **Gastrointestinal Drugs** | **263** |  |
| **Central Nervous System Agents** | **68** |  | *PPI* | *152* | 58% |
| *Antihistamines* | *26* | 38% | *Symptomatic agents* | *12* | 5% |
| *SSRI/SNRI* | *9* | 13% | *Anti-nausea/vomiting* | *37* | 14% |
| *Anti-seizure* | *12* | 18% | *Laxatives* | *57* | 22% |
| *Anxiolytics* | *9* | 13% | *Miscellaneous* | *5* | 2% |
| *Antipsychotics* | *5* | 7% | **Medication reconciliation/Education** | **201** |  |
| *Miscellaneous* | *7* | 10% | **Non-pharmacological** | **103** |  |
| **Diagnostics** | **448** |  | *Lifestyle modification* | *98* | 95% |
| *Lab tests* | *337* | 75% | *VTE prophylaxis* | *5* | 5% |
| *Electrolytes* | *54* | 12% | **Analgesics and Anti-inflammatory agents** | **381** |  |
| *Medication management* | *24* | 5% | *Paracetamol* | *203* | 53% |
| *Physicals* | *16* | 4% | *NSAIDs* | *135* | 35% |
| *Miscellaneous* | *17* | 4% | *Opioids* | *39* | 10% |
| **Endocrine System and Hormonal Agents** | **486** |  | *Miscellaneous* | *4* | 1% |
| *Anti-Diabetics* | *452* | 93% | **Respiratory Tract Agents** | **29** |  |
| *Hormonal agents* | *24* | 5% | **Urinary-Tract Disorders Agents** | **22** |  |
| *Steroids* | *9* | 2% | **Vitamins and Nutritional Agents** | **223** |  |
| *Miscellaneous* | *1* | 0% | **Miscellaneous** | **58** |  |

| **Table S2. Clinical examples of intervention severity** | | | | |
| --- | --- | --- | --- | --- |
| **Negligible** | **Minor** | **Moderate** | **Major** | **Catastrophic** |
| Pain score issues | Home meds discrepancy | Modify dose/frequency of medications (excluding antibiotics and anticoagulation) | Management of hypoglycemia | Prevent ICU admission |
|  | Lifestyle modification | Perioperative medication management | Adjustment of antibiotic dose or frequency according to kidney function | Discontinue a teratogenic medication in a pregnant patient |
|  | Patient education | Management of chronic conditions or inpatient complications | Alternative therapies according to kidney function (e.g. NSAIDs) | Preventing VTE |
|  | Duplicate order | Discontinue not indicated medications | Antibiotic dose calculation (e.g. colistin) |  |
|  | Non formulary to formulary selection | Request labs | Escalation/de-escalation of antibiotic according to culture and clinical improvement |  |
|  |  | Respond to drug information inquiry | Anticoagulation perioperative management |  |
|  |  |  | Adding new therapy based upon new diagnosis (e.g. diabetes, hypertension, dyslipidemia) |  |
| NSAIDs: nonsteroidal anti-inflammatory drugs; ICU: intensive care unit; VTE: venous thromboembolism | | | | |
